# Neuromorphic Stereo Vision: A Survey of Bio-Inspired Sensors and Algorithms

**DOI:** 10.3389/fnbot.2019.00028

**Published:** 2019-05-28

**Authors:** Lea Steffen, Daniel Reichard, Jakob Weinland, Jacques Kaiser, Arne Roennau, Rüdiger Dillmann

**Affiliations:** ^1^FZI Research Center for Information Technology, Karlsruhe, Germany; ^2^Humanoids and Intelligence Systems Lab, Karlsruhe Institute of Technology (KIT), Karlsruhe, Germany

**Keywords:** bio-inspired 3D-perception, neuromorphic visual sensors, cooperative algorithms, event-based technologies, brain-inspired robotics, human-like vision

## Abstract

Any visual sensor, whether artificial or biological, maps the 3D-world on a 2D-representation. The missing dimension is depth and most species use stereo vision to recover it. Stereo vision implies multiple perspectives and matching, hence it obtains depth from a pair of images. Algorithms for stereo vision are also used prosperously in robotics. Although, biological systems seem to compute disparities effortless, artificial methods suffer from high energy demands and latency. The crucial part is the correspondence problem; finding the matching points of two images. The development of event-based cameras, inspired by the retina, enables the exploitation of an additional physical constraint—time. Due to their asynchronous course of operation, considering the precise occurrence of spikes, Spiking Neural Networks take advantage of this constraint. In this work, we investigate sensors and algorithms for event-based stereo vision leading to more biologically plausible robots. Hereby, we focus mainly on binocular stereo vision.

## 1. Introduction

As the visual sense and any visual sensor loose one dimension when mapping the 3D-world onto a 2D-representation, the ability to recover depth is crucial for biological and artificial vision systems. Stereo-vision refers to the method recovering depth information from both eyes, or in the artificial context, two sensors. In biology this is possible due to the laterally shifted eyes, gaining slightly different versions of a scene. The brain matches the corresponding points of both images and computes their disparity.

While biology computes disparities seemingly effortless, current approaches computing stereo in real-time are too computationally expensive. This is mainly caused by acquiring and processing huge amounts of redundant data. Hence, frame-based data acquisition implies computational limitations (Rogister et al., 2012). Furthermore, with increasing complex scenes and noise the computational expense of common machine vision system increases significantly. That has negative effects on the speed, size, and efficiency of the hardware (Osswald et al., 2017). Finding the corresponding dots in both images is hereby the bottleneck. This computationally complex issue is referred to as the correspondence problem. With the development of neuromorphic visual sensors (Lichtsteiner et al., 2008), a new physical constraint is now also applicable in artificial vision: time (Kogler et al., 2011a; Rogister et al., 2012; Dikov et al., 2017). Similar to retinal output cells, event-based sensors transmit information asynchronously as a continuous stream of events (Rogister et al., 2012). A comprehensive scientific investigation of the neural code of the retina is provided in Meister and Berry (1999).

Spiking Neural Networks are a natural match for event-based sensors due to their asynchronous operation principle. Thus, SNNs are a popular choice for many systems using silicon retinas like the work of Orchard et al. (2013), Orchard et al. (2015), and Haessig et al. (2017). Examples for event-based stereo vision applications applying networks with spiking neurons are Dikov et al. (2017), Osswald et al. (2017), Rebecq et al. (2017), and Haessig et al. (2019).

As self-driving cars are already a very promising application of artificial depth perception, they are also an interesting field of use for event-based 3D-vision. An approach combining event-based vision and deep learning for steering prediction for autonomous vehicles is introduced in Maqueda et al. (2018). Furthermore, event-based vision is changing technologies and algorithms in fields such as health-care, security, surveillance, entertainment and industrial automation (Brandli, 2015). In Mafrica (2016), EBS for robotic and automotive applications are investigated.

Scientists in the field of computer vision and 3D-imaging strive for the sophisticated model posed by nature. Nevertheless, a comprehensive review, not only about human inspired sensors but also biologically plausible algorithms and the synergy of both, is still missing. This paper surveys the advances of event-based techniques and algorithms, especially developed for neuromorphic visual sensors, researchers have made to this day. As stereo vision is a large topic many different techniques such as radar, ultrasonic sensors, light section, structured light, and depth from defocus/focus exist. However, this manuscript is mainly focusing on binocular stereo vision.

For this purpose, conventional machine stereo vision is reviewed briefly and vision in nature is elaborated in more depth. Subsequently, the evolution and a comparison of event-based sensors is presented, followed by an investigation of cooperative algorithms and their alternatives for event-driven stereo vision.

## 2. Technical and Biological Background

Machine stereo vision, also referred to as stereoscopic vision, has been an active field of research for decades. It has been widely investigated before the arise of event-based sensors. However, biology understands a scene faster than computers and at lower energy budget (Martin et al., 2018). It works reliable in human vision and error robustness and energy efficiency are sophisticated. Hence, nature can be used as an inspiration for more efficient and robust sensors and algorithms. This section covers standard cameras and their mechanics as well as the human retina and depth perception in nature.

### 2.1. Conventional Cameras and Their Principle of Operation

Customary cameras commonly use a sensor constituted of a 2D-Array of pixels, where each pixel is sensitive to light intensity. Data is selected synchronously from all pixels at fixed time steps. The generated pixel data at one time is called frame and thus the frequency of the read-out is called frame rate (Mahowald, 1992; Akolkar et al., 2015). These sensors are limited in their performance by their course of action. Imaging and information transfer at a fixed frame rate, unrelated to the dynamics of the observed scene, causes two opposed issues. For one, important information might get lost leading to a decrease in temporal resolution. This is less crucial but still true for relatively high frame rates, since events might always occur between these two time steps. The complementary problem is an inevitably high redundancy. Data transfer of all pixels, even in case of no or small local changes, increase the data transfer and volume needlessly. This problem is magnified as changes usually only affect a small part of the scene, like a subset of pixels, and rarely the whole image (Posch et al., 2014).

### 2.2. Depth Perception in Machine Vision

There are a lot of techniques to obtain 3D-data of a scene. Active representatives are electro-optical distance measurements such as LIDAR (light detection and ranging), TOF cameras (time-of-flight), radar, ultrasonic sensors, light section, and structured light. In addition there are passive techniques such as SfM (Structure from motion), shape from shading and stereopsis. However, most of these technologies are slow, computation-intensive and resource-gobbling. In case of LIDAR the 3D-generation itself is rather cheap but it outputs a lot of points that are expensive to handle. These drawbacks are problematic for many applications using 3D-data, like navigation, path planning, and robotic motion control. Cameras are a good option because they produce dense data in real time. However, since cameras represent the 3D-environment in 2D-data, depth information must be gained supplementary. The obvious way is stereoscopy, a form of sensor fusion in which the same scene is recorded from at least two different points of view and the data is combined into a sole representation. If you have the matching dots from both images, the depth can be determined since it is inversely proportional to the disparity. Disparity is the displacement along the epipolar line.

**Figure 1:**
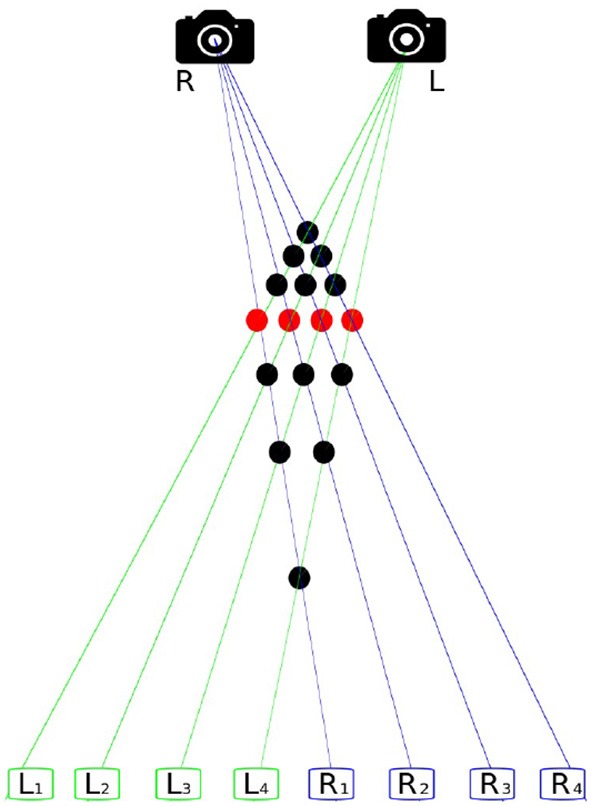
The correspondence problem. The scene comprises four identical objects, recorded from two perspectives. *R* is the right and *L* the left camera. The rectangles *L*_1_–*L*_4_ represent the imaging of *L* and *R*_1_–*R*_4_ of *R*. There are 16 possible matches, visualized by dots, showing how the shots of *L* and *R* might correspond to each other. Only four of them, accentuated in red, are correct matches.

According to Lucas and Kanade (1981) the 3D-position of an object is reconstructable if enough of its dots can be found and matched from at least two images, taken from slightly differing perspectives. This requires four steps; (1) finding of objects and features in the image, (2) matching the points found, (3) estimating the camera parameter, and (4) determining the distance from the camera objects represented by the dots. This process is called image registration and the basic problem of finding points that belong together in two images of the same scene is called the correspondence problem. Figure 1 shows why this problem is not trivial. In this case the solution is particularly difficult because the four depicted objects are indistinguishable. Hence, further methods are necessary to determine correct correspondences.

Stereo vision is a very well-investigated research area in the field of machine vision. In Marr and Poggio (1976), the authors laid the foundation for research in this field at an early stage. In Barnard and Fischler (1982), Dhond and Aggarwal (1989), and Scharstein et al. (2002) different approaches to overcome the stereo correspondence problem are presented and in Scharstein et al. (2002) a general taxonomy is proposed two-frame stereo methods regarding comparison of multi-view 3D-reconstruction methods, differentiating their key properties. In Seitz et al. (2006), this In Seitz et al. (2006), this taxonomy is expanded and refined. On this basis six algorithms (Kolmogorov and Zabih, 2002; Pons et al., 2005; Goesele et al., 2006; Vogiatzis et al., 2007; Furukawa, 2008) for reconstruction of dense objects with calibrated cameras are calibrated cameras are evaluated. The authors of Seitz et al. (2006) measure accuracy (how close the reconstruction truth model) and completeness (how much of the ground truth model is successfully reconstructed) of all methods to provide a good comparison. It is stated that except (Goesele et al., 2006), all evaluated techniques are complete. evaluated techniques are complete. Hernández Esteban and Schmitt (2004) achieves the highest accuracy, with 90% of its ground truth mesh. It is also worth mentioning, that the runtimes vary drastically. The fastest approach is drastically. The fastest approach is Pons et al. (2005) and the slowest one is Goesele et al. (2006). A quite general review about the broad range of 3D-reconstruction techniques, is provided in Butime et al. (2006). Here, the camera-based approaches.

Methods for artificial stereoscopy can be divided into two groups, sparse and dense scene representation. Sparse approaches include especially early, often feature-based, work. Many of those use edge detectors or interest operators to detect promising areas of the image and find their correspondences. Newer approaches from this area extract very reliable characteristics and use them as seeds to determine further correspondences (Szeliski, 2010). The second group, dense methods, although more complex, are more popular nowadays. In Scharstein et al. (2002), a taxonomy for these approaches is presented, defining the four steps, (1) matching cost computation, (2) cost (support) aggregation, (3) disparity computation/optimization, and (4) disparity refinement as the basis of such algorithms. Most of the approaches in this group can be subdivided into these sections, although a subgroup of these points can already form a full-fledged algorithm. A further differentiation results in local and global methods (Szeliski, 2010). With the local approach only intensity values within a finite range are considered for the calculation of the disparities of a point. Many local algorithms, such as the sum-of-squared-differences (SSD), consist of steps 1–3, but a few consist only of steps 1 & 2. In contrast, global methods are based on smoothness assumptions and usually refer to the entire image. They usually do not use aggregation and often consist of steps 1, 3, & 4. To optimize the outcome simulated annealing, expectation maximization or graph cuts are often applied. Additionally to global and local methods there are also iterative algorithms (Scharstein et al., 2002; Szeliski, 2010) including the biologically motivated approach of Marr and Poggio (1976). In the case of increasingly complex scenes and in the case of noisy image data, the classical approaches for stereoscopic vision quickly reach their limits and also the computational effort is disproportionately large. This has a huge impact on the size, speed, power consumption, throughput, and efficiency of the hardware used and makes their integration difficult (Osswald et al., 2017).

### 2.3. The Retina

**Figure 2:**
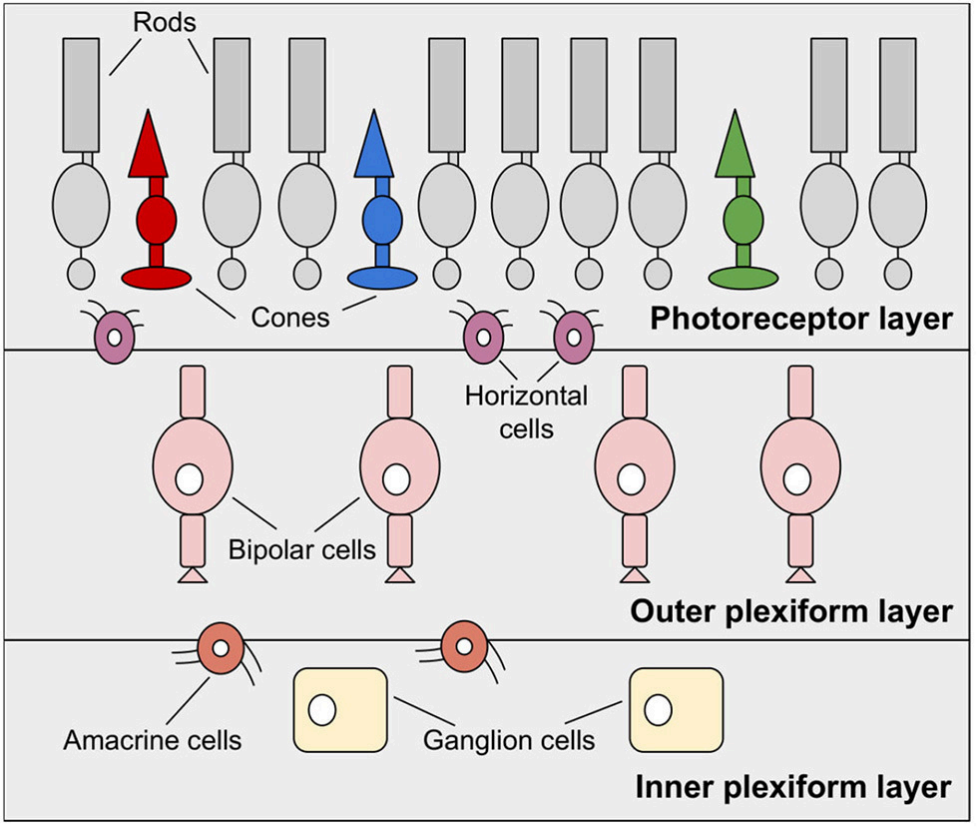
The human retina, reduced to essential layers for neuromorphic visual sensors. The photoreceptor layer, the outer plexiform layer including bipolar cells and the inner plexiform layer made up of ganglion cells. Additionally, horizontal cells and amacrine cells connect these layers.

The retina, also known as the fundus, is a highly developed system consisting of photosensitive cells that contain approximately 100 million black-and-white photoreceptors and nearly 4 million color receptors (Boahen, 1996). It is a multi-layered neuronal network responsible for the acquisition and preprocessing of visual information. As shown in Figure 2 the retina is divided into three main layers, the photoreceptor layer, the outer plexiform layer, and the inner plexiform layer (Posch et al., 2014). These layers include, with the photoreceptors, the bipolar cells, and the ganglion cells, the three most important cell types.

Photoreceptors are the actually light-sensitive cell type of the retina and can be divided into two types that react to different wavelengths of light. Cones for color recognition and sharp vision, as well as rods for vision under bad lighting conditions (Rodieck, 1998). These sensory cells convert incident light into an electrical signal which influences the release of neurotransmitters and thus triggers a chain reaction (Posch et al., 2014). In darkness, the non-excited normal state, photoreceptors secrete neurotransmitter exciting the bipolar cells. Subsequently, the stimulated bipolar cells also release neurotransmitters inhibiting the ganglion cells. This means that when no light penetrates the eye, photoreceptors, and bipolar cells are active and ganglion cells are inactive. If the illumination increases significantly, the depicted process drives the ganglion cells creating action potentials that reach the visual center of the brain via the optic nerve (Ganong, 1972; Goldstein, 2015).

The sensory cells of the outer as well as the inner plexiform layer, by name the bipolar cells and the ganglion cells, can be divided into *ON-* and *OFF-types*. The ON-bipolar cells code for bright and the OFF-bipolar cells for dark time-space differences. In the absence of a stimulus both cells generate a few random spikes. However, if the illumination is increasing, the ON-cell increases its firing rate when not stimulated while the OFF-cell no longer generates any pulses at all. In the case of a negative change in illumination, if it gets darker, this effect reverses (Rodieck, 1998). This effect is achieved by comparing individual signals of the photoreceptors with time-space average values, determined by means of horizontal cells. Horizontal cells interconnect photoreceptors and bipolar cells laterally. Respectively, the diverse amacrine cells mediate signal transmission between bipolar cells and ganglion cells (Posch et al., 2014). Amacrine cells are inhibitory interneurons and therefore regulate other cells by repression. There are at least 33 subgroups which are mainly characterized by their diameter and thus in their sphere of influence. The smallest variety, narrow-field amacrine cell (NA), is only about 70 *μ*m in diameter. In addition, there are medium-field (MA), with about 70 *μ*m, and wide-field amacrine cells (WA), with about 350 *μ*m diameter (Balasubramanian and Gan, 2014).

1. **Local automatic gain control (*DP 1*)** at the photoreceptor and network level is the preprocessing by means of time-space bandpass filtering and adaptive sampling. As a result, the receptors are independent of absolute values and instead measure the changes of illumination with an adaptive accuracy. This leads to a larger dynamic range of the input without increasing the output unnecessarily. The dynamic range is defined as the ratio between maximum processable signal and background noise in darkness (Posch et al., 2011).
2. **Bandpass spatio-temporal filtering (*DP 2*)** in the outer plexiform layer limits the frequencies in both directions. By suppressing low frequencies, redundancies are discarded and inhibiting high frequencies reduces noise in moving objects. In addition, high-pass filters of the inner plexiform layer emphasize this effect.
3. **The equalization of the signal (*DP 3*)** by means of ON- and OFF-types lowers the spike rate. Without this separation, a significantly higher coding rate would be required in order to encode positive and negative values on one channel.
4. **High spatial and temporal resolution (*DP 4*)** of the entire signal is simulated by the distribution of sustainable parvocellular cells (P-cells) and the volatile magnocellular cells (M-cells) in the retina. In fact, in the center of the visual field the spatial resolution is high and the temporal resolution low. At the edge region it is the other way around. The effect is further enhanced by precise fast eye movement.

DP 1 & DP 2 are implemented in the outer and DP 3 & DP 4 in the inner retinal layer Posch et al. (2014). The retina is responsible for converting spatio-temporal illumination information into pulses. This information is then transmitted via the optical nerve to the visual cortex. The four design principles, above all adaptive filtering and sampling, allow flexible, high-quality signal processing, and efficient coding maximizing the information content (Boahen, 1996, 2000; Posch et al., 2014).

### 2.4. Biological Depth Perception

In biological imaging, the 3D-environment is projected onto a 2D-representation and thus the precise position of objects in space is lost. Safe navigation in unknown surroundings, as well as the estimation of distances is only possible for humans, because we can reconstruct depth from 2D-information and are thus capable of 3D-perception.

An important part for depth perception, is *a priori knowledge*. That refers to the ability of humans to consider past stimuli. Hence, the brain can see 3D even if it is not receiving any depth information just now, but did so a second ago. The principle is similar to the core idea of event-based vision; to not acquire the current depth, only changes in local depth.

Apart from *a priori knowledge*, the techniques for depth perception can be roughly divided into oculomotor and visual stimuli (Ganong, 1972; Goldstein, 2015). For oculomotor depth criteria, also referred to as oculomotor cues the position of the eyes and the tension of the eye muscles are decisive. The eye position is used to measure the distance of the focused object. In addition, the muscles are tense for near objects and relaxed for distant ones. Oculomotor cues are useful for vision at close range until approximately one arm's length from the eye and are created in two different ways. The first is the convergence (up to 600 cm) resulting from the movement of the eyes toward the center, when objects located nearby, are observed. On the other hand it concerns accommodation (20–300 cm) caused by the change in shape of the eye lens when objects at different distances are focused (Ganong, 1972; Cutting, 1997).

Visual depth criteria are further divided monocular and binocular vision. Monocular refers to all depth information that can be obtained by one eye alone, and binocular means that two eyes are required. Monocular vision therefore refers to all the information we can extract from a simple 2D-signal in order to understand a scene. For one this concerns static monocular cues like the knowledge about the common shape and size of known objects, as well as texture and shadow as well as the fact that people can segment objects well on the basis of context. Furthermore, based on the perspective and scene continuity and the assumption that objects either stay in place or move according to physical laws we can derive more information. In addition to static, there is dynamic monocular vision. It is created by movement-induced depth stimuli, which are produced by head and eye movements. This includes the covering and uncovering of objects as well as the parallax. The latter occurs when several objects are located at different distances from the observer, who is moving parallel to them. The near objects move, in the perspective of the observer faster than the more distant ones (Ganong, 1972; Cutting, 1997; Goldstein, 2015).

According to Rose (1980), binocular sensitivity is higher than monocular sensitivity. Additionally, a comparison of monocular and binocular stimuli states that binocular have shorter latencies than monocular responses (Adachi-Usami and Lehmann, 1983). Binocular vision distinguishes between simultaneous vision, fusion and stereopsis. Under simultaneous vision one understands that certain visual impressions are captured by both eyes simultaneously. This serves for the suppression of false visual sensation caused for instance by illnesses related to strabismus. The dual perception of simultaneous vision helps to avoid disturbing effects. The merging of the two separately recorded signals of both eyes, is named fusion and it is necessary to not permanently see double (Ganong, 1972; Goldstein, 2015). The basis of stereopsis is disparity and is caused by the fact that, although both visual fields overlap for the most part, corresponding points differ slightly due to a view angle shifted by ~6 cm. Disparity, the horizontal displacement, is inversely proportional to the depth. This coherence is the basis of the correspondence problem in binocular vision shown graphically in Figure 1 (Julesz, 1960; Ganong, 1972; Goldstein, 2015). Julesz showed by means of a simple experiment how our brain can reliably solve the correspondence problem (Julesz, 1960, 1964). For this purpose, random dot diagrams were shown to a group of participants. He used two graphics of dots which are identical except for a slightly shifted square in the center. The participants were able to see a depth map representing the offset square. This effect occurs because the brain tries to reconcile both signals, but there is a difference in elevation. Research based on random dot diagrams led to one of the most influential books (Julesz, 1971) in cognitive sciences and the basic work for stereo vision. How exactly the brain establishes the connection between two points of the retina, and thus solves the correspondence problem, is still an active field of research. In Cumming and Parker (1997), theories are investigated to what extent the signals of cortical neurons are related to conscious binocular depth perception.

## 3. Event-Based Visual Sensors

In Delbrück et al. (2010), the utopian features of a perfect camera are identified as an infinitely high resolution, an infinitely wide contrast range and an infinite number of frames per second. At the same time, this ideal sensor has a pixel size of zero and effectively no power consumption. Since nature is much closer to this ideal than conventional cameras, scientists started to imitate biological systems. Biologically inspired camera systems absorb light just like their biological counterparts. In biology, photoreceptors are used therefore and for the artificial afterimage electrical circuits containing photodiodes are applied. The data processing of such a perfect sensor would of course be enormously computationally demanding, but nature also has a solution for this; retinas take over a large part of the processing and thus only transmit relevant information to the brain (Delbrück et al., 2010). Artificial retinas also take this aspect into account. They acquire and transmit information according to the dynamics of the recorded scene. They are asynchronous in their course of transmission, and therefore do not output a fixed number of data packets per second. Instead they broadcast information independently for individual pixels if their illumination changes significantly. This is a great derivation from the rigid control and transmission mechanisms of conventional sensors and implies considerable advantages for many applications. Particularly worth mentioning is the latency in the microsecond range, the extremely high contrast range and the avoidance of motion blur. A profound survey of neuromorphic sensors is given in Posch et al. (2014). Hence, section 3.1 refers to this work to some extent.

Spiking Neural Networks (SNN) are members of the family of Artificial Neural Networks (ANN) but spiking neurons provide a closer and more accurate model of biological neurons. The unique characteristic of SNNs is continuous input over time and they are referred to as the third generation of ANNs. For a comprehensive introduction to SNNs see Maass (1997), Vreeken (2003), and Grüning and Bohte (2014). However, their asynchronous principle of operation is perfectly suited for processing even-based data, as the natural output of EBS is the required form of input for SNNs. A biological introduction, and a survey of how SNNs can be used for robotics is given in Bing et al. (2018). A discussion of the advantages of combining EBS and SNN is done in Akolkar et al. (2015).

However, as parallelism is a key component of EBS and SNN, they require dedicated hardware to run efficiently. Neuromorphic hardware as SpiNNaker (Furber et al., 2006, 2014), ThrueNorth (Merolla et al., 2014), Spikey (Pfeil et al., 2013), and Loihi (Davies et al., 2018) model the massively parallel structure of the brain. Algorithms including spike and event-based communication often enhance their performance when run on neuromorphic hardware. Energy efficiency, scalability, and real-time interfacing with the environment caused by high parallelism are advantages of this technology (Furber et al., 2014; Davies et al., 2018). Furthermore, fault tolerance is a huge benefit of this brain inspired hardware. Much like neural structures in nature, neuromorphic systems cope well with the failure of single components.

In the field of machine learning, it was shown several times that neuromorphic hardware can be applied successfully to biologically inspired algorithms. For instance, in Neftci et al. (2014) a Restricted Boltzmann Machine using leaky integrate-and-fire neurons with STDP synapses is used for learning a generative model of the MNIST dataset of hand-written digits. Another example is shown in Bogdan et al. (2018). The authors implement a technique for topographic map formation on SpiNNaker. Eventhough stereo vision applications have not been implemented on neuromorphic systems a lot. Although first approaches like (Dikov et al., 2017; Andreopoulos et al., 2018) exist, implying that there is potential. Furthermore, running event-based stereo vision algorithms on neuromorphic hardware creates a complete event-based chain from the data acquisition to the processing.

### 3.1. The Silicon Retina—Emergence and Fundamentals

**Figure 3:**
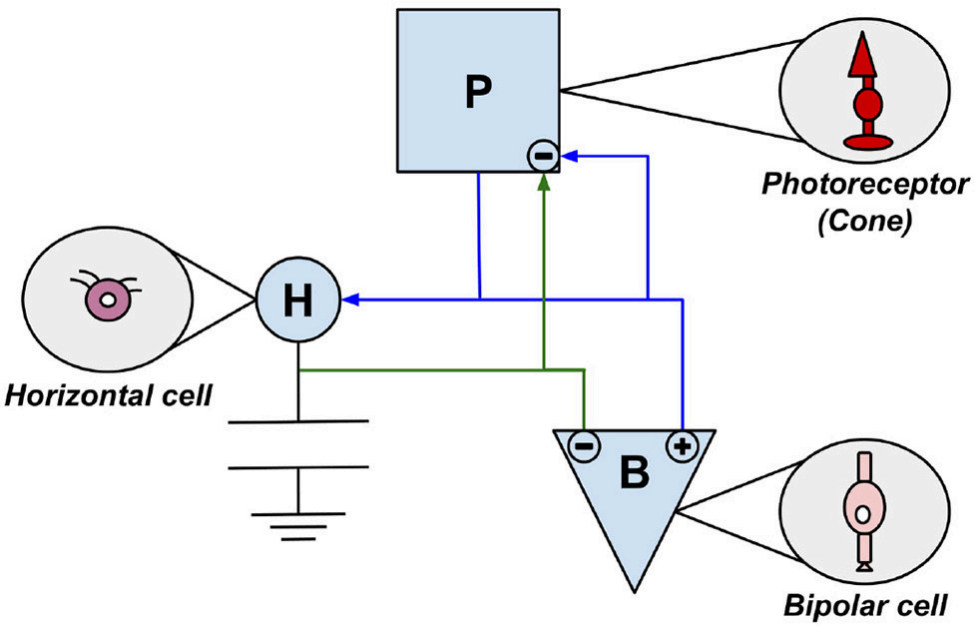
The three components of Mahowalds silicon retina are modeled on photoreceptor cells, bipolar cells, and horizontal cells. Every module is marked by the first letter of its biological paragon.

Over the last 50 years, scientists have developed visual sensors, so-called silicon retinas, modeled after the biological retina and thus employing neurobiological principles. Many of the technologies developed are based on the principles of *very large scale integration(VLSI)*. Pioneers for silicon retinas are Mahowald and Mead who had already introduced their *Silicon VLSI Retina* in 1991 (Mahowald and Mead, 1991; Mahowald, 1994). This sensor has adaptable photoreceptors and a network capable of spatial smoothing (Lichtsteiner et al., 2008). It is a sensor chip with a 2D hexagonal grid of pixels. In this sensor they replicated some cell types of biological retinas. This concerns the photoreceptors, bipolar cells and horizontal cells discussed in chapter 2.3. The interaction of the three components and their affiliation to their biological model is visualized in Figure 3. The artificial photoreceptor (P) is modeled based on the cone and consists of two components, a time-continuous light sensor and an adaptive circuit (Mahowald and Mead, 1991; Mahowald, 1992; Douglas et al., 1995; Posch et al., 2014).

The layer of horizontal cells (H) located between the photoreceptor layer and the outer plexiform layer (see Figure 2) is represented by a network of adjustable MOS resistors (Posch et al., 2014). The circuits representing bipolar cells (B) amplify differences between the values measured by P and the local average. The component B additionally converts these signals into ON- and OFF-values (Mahowald, 1994; Posch et al., 2014). Since this sensor represents merely the photoreceptor layer, the outer plexiform layer and their connecting layer, thus the inner layers of the retina, only DP 3 & DP 4 from chapter 2.3 are converted. This sensor was used exclusively for test and demonstration purposes proofing biological theses (Lichtsteiner et al., 2008).

In contrast, the *Parvo-Magno Retina* by Zaghloul and Boahen considers five retinal layers. It comprises the three main layers shown in Figure 2 and both intermediate layers of horizontal and amacrine cells (Boahen, 2005; Zaghloul and Boahen, 2006). This technology emphasizes the realistic imitation of P-cells (sustainable parvocellular cells) and M-cells (volatile magnocellular cells) of both plexiform layers. The Parvo-Magno Retina is superior to the Silicon VLSI Retina by the implementation of the outer retinal layers. In addition to DP 3 & DP4, it implements two further properties of biological retinas: adaptation to lighting conditions and local contrast (see DP 1 in chapter 2.3) and flexible spatio-temporal filtering (see DP 2 in chapter 2.3).

**Figure 4:**
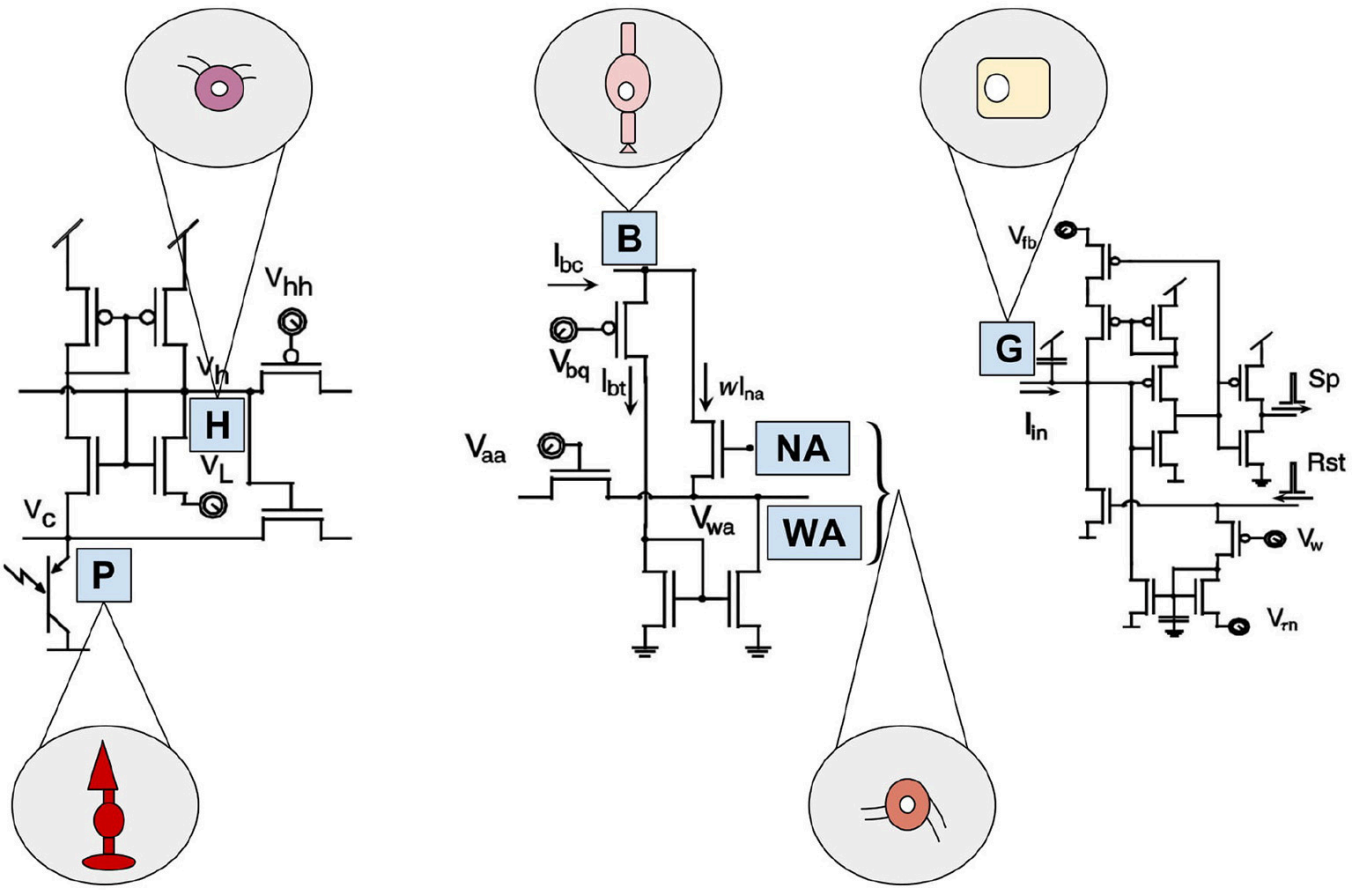
Artificial building blocks and their biological models of the Parvo-Magno Retina from Zaghloul and Boahen. The left circuit shows the outer retinal layer. A phototransistor takes current via an nMOS transistor. Its source is connected to *V*_*c*_, representing the biological photoreceptor (P). Its gate is connected to *V*_*h*_, portraying horizontal cells (H). The circuit in the center represents the amacrine cell modulation. A bipolar terminal B excites a network of wide-field amacrine cells (WA) and also narrow-field amacrine cells (NA) through a current mirror. The amacrine cells, in turn have an inhibitory effect on B. By the right circuit the spiking ganglion cells are represented. Current *I*_*i*_*n* from the inner retinal circuit charges up a membrane capacitor G, based on biological ganglion cells. If its membrane voltage crosses a threshold a spike (Sp) is emitted and a reset (Rst) discharges the membrane. Inspired by Zaghloul and Boahen (2006).

Despite its promising structure, the Parvo-Magno Retina from Zaghloul and Boahen is difficult to apply for practical use-cases. This is mainly due to the lack of correspondences between the response characteristics of the pixels (Posch et al., 2014). This concerns strongly fluctuating spike rates of the pixels as well as many non-sensitive pixels which do not react even with comparatively high stimuli (contrast up to 50%) (Lichtsteiner et al., 2008). However, this feature does not mark down this sensor compared to other models of its time. Many early representatives of the silicon retina are not suitable for any real applications. This is mostly down to the fact that their developers were mainly biologists rather than engineers and their motivation was to verify neurobiological models. Common weaknesses of these technologies are an extremely complex circuit (see Figure 4), a large retinal area and a low filling factor. On top of that they are susceptible to noise and VLSI implementations tend to have device conflicts. These issues prevented their use in practice so far (Posch et al., 2014). A few years ago, however, there was a turnaround. More and more developers with a technical background, practice-oriented motivation and the necessary knowledge, became involved. The scientific team around Ruedi developed one of the first sensors with a stronger focus on applicability (Ruedi et al., 2003). His team focused mainly on spatial and only subordinately on temporal contrast. After a period of global integration the system weighted events according to the strength of their spatial contrast. This implies a big advantage since events with high contrast are prioritized for transmission during a period of high data throughput. This ensures that despite limited bandwidth and high data volumes, no important information will be lost. The sensor is characterized by a large contrast range, but suffers greatly from temporal redundancies and a temporal resolution which, due to global integration, is limited by the frame rate (Lichtsteiner et al., 2008).

The approach of Mallik et al. (2005) goes in a similar direction. Here, too, typical event-based technologies, such as the communication protocol presented in chapter 3.2, are used for synchronous image acquisition. The active pixel sensor (APS) CMOS is modified in such a way that absolute exposure changes are detected. The advantages of an APS with small pixels are put into perspective by the small contrast range and the absolute exposure measurement. Therefore, good results can only be achieved with uniform illumination of the scene (Lichtsteiner et al., 2008).

The sensors of Ruedi et al. (2003) as well as Mallik et al. (2005) are, regarding their technical implementation, far superior to the cameras of Mahowald and Mead (1991) and Boahen (2000). What they gain in practical applicability, however, they lose in biological plausibility, mainly due to their synchronous mode of action.

Today's representatives of the silicon retina, represent a compromise of biological and technical aspects. They implement all the design principles of biological retinas presented in chapter 2.3 at the pixel level, as did the Parvo-Magno retina. This concerns local gain control (1.DP: amplification), pre-processing by spatio-temporal bandpass filtering (2.DP: processing), adaptive sampling (3.DP: detection), and continuous perception (4.DP: quantification) (Boahen, 1996). Delbrück and Posch are to be emphasized on their technical achievements. Their work is discussed in more detail in Chapter 3.3 (Lichtsteiner et al., 2008; Liu and Delbrück, 2010; Chen et al., 2011; Posch et al., 2011).

In Delbrück et al. (2010) criteria for the classification of biologically inspired sensors are introduced. These are summarized in Table 1. Sensors that fall under the category *spatial contrast (SC)* instead of *spatial difference (SD)* can handle temporal variations with regard to scene lighting better. This is because the use of relative intensity ratios instead of absolute intensity differences suppresses spatial redundancies. Respectively, cameras from the category *temporal contrast (TC)*, compared to *temporal difference (TD)*, are better in respect to dealing with uneven, spatially varying lighting conditions. The reason for this is that relative instead of absolute intensity changes are considered and thus temporal redundancies are suppressed. This criteria is also applied in Table 2 of chapter 3.3, the comparison of current event-based sensors.

**Table 1:** Three criterions to classify EBS.

**Criterion**	**Name**	**Benefits**
Spatial	Spatial contrast (*SC*)	Reducing spatial redundancies makes it well suited for unsteady lighting conditions.
	Spatial difference (*SD*)	Cheap
Temporal	Temporal contrast (*TC*)	Reducing temporal redundancies makes it well suited for uneven lighting conditions.
	Temporal difference (*TD*)	Easy to implement
Data acquisition	Frame event (*FE*)	Cheap hardware and easy to implement
	Asynchronous event (*AE*)	Low latency, requires relatively few computing power

*The categories (Spatial) and (Temporal) differentiate between relative contrast and absolute differences. Its flexible and adaptive nature makes relative contrast beneficial in case of unsteady and uneven lightning conditions*.

**Table 2:** Comparison of event-based sensors.

	**DVS**	**DAVIS**	**ATIS**
Major function	Asynchronous detection of temporal contrast	See DVS + synchronuos imaging	See DVS + Intensity measurement for every single event
Resolution	128 × 128	240 × 180	304 × 240
Gray-scale value	**✗**	Synchronous	Asynchronous
Circuits per pixel	1	1	2
Exposure time	**✗**	Uniform	Uneven
Latency	15 *μ*s	3 *μ*s	4 *μ*s
Noise	Very strong (2.1%)	Strong (APS: 0.4%, DVS: 3.5%)	Medium (0.25%)
Dynamic range	120 dB	130 dB	143 dB
Pixel size	0.35 × 0.35 *μ*m	0.18 × 0.18 *μ*m	0.30 × 0.30 *μ*m
Costs	2,590/2,250 €	4,140/3,630 €	5,000/4,000 €
Contrast sensitivity	15%	11%	30%
Date of publication	2008	2013	2011
Application	Dynamic scenes	Dynamic scenes	Surveillance
Classification regarding Table 1			
	SC TD AE	SC TD APS: FE DVS: AE	SC TD & TC AE

*As costs the standard and the reduced costs for scientific and educational purpose is listed. Information originates from Posch et al. (2014), Dong-il and Tae-jae (2015), and Cohen et al. (2017)*.

The four design principles of biological vision and their implementation in conventional and event-based cameras is as follows:

- **Amplification:** While automatic gain control is global in conventional cameras it is realized locally in the retina.
- **Preprocessing:** Preprocessing is not applied in standard sensors but the retina uses band-pass filters.
- **Detection:** Since standard cameras make use of integrating detectors, such as CCD, resets are often required.
- **Quantization:** While fixed in standard cameras in retinas quantization is adaptable to the frequency of change and the distribution of the input signal. Event-based sensors privilege time, while classic cameras privilege precise pixel intensity.

The main distinguishing features are the lack of frames, low latency, low processing power and a high contrast range.

### 3.2. Address Event Representation

In nature, data transfer from the eye to the brain is carried out by approximately one million axons of ganglion cells. For a realistic technical imitation, each pixel of the camera needed its own cable. Since any practical chip wiring makes this impossible, VLSI-technologies employ a workaround (Posch et al., 2014).

To bundle the data traffic on these lines, an event-based data protocol Address Event Representation (AER) is used. The research and development that is leading to this technology was largely pioneered in the late 80s by Sivilotti (1990) and Mahowald (1992). Both scientists were part of the Caltech group of Carver Mead. AER is an event-controlled, asynchronous point-to-point communication protocol for neuromorphic systems. It allows spikes to be transferred from neurons of one chip to neurons of a second chip (Boahen, 1998, 2000). The basic idea is based on the addressing of pixels or neurons, with their *x-* and *y-value*, within their array (Lichtsteiner et al., 2008).

For a long time, this technology has only been used by a small group of researchers, such as Boahen (1996) for prototypes of the Silicon Retina. It was not until after the turn of the millennium that a broader public took notice of it. In addition to the development of many more biological camera systems, AER also found its way into other contexts, such as biological hearing and wireless networking (Posch et al., 2014).

**Figure 5:**
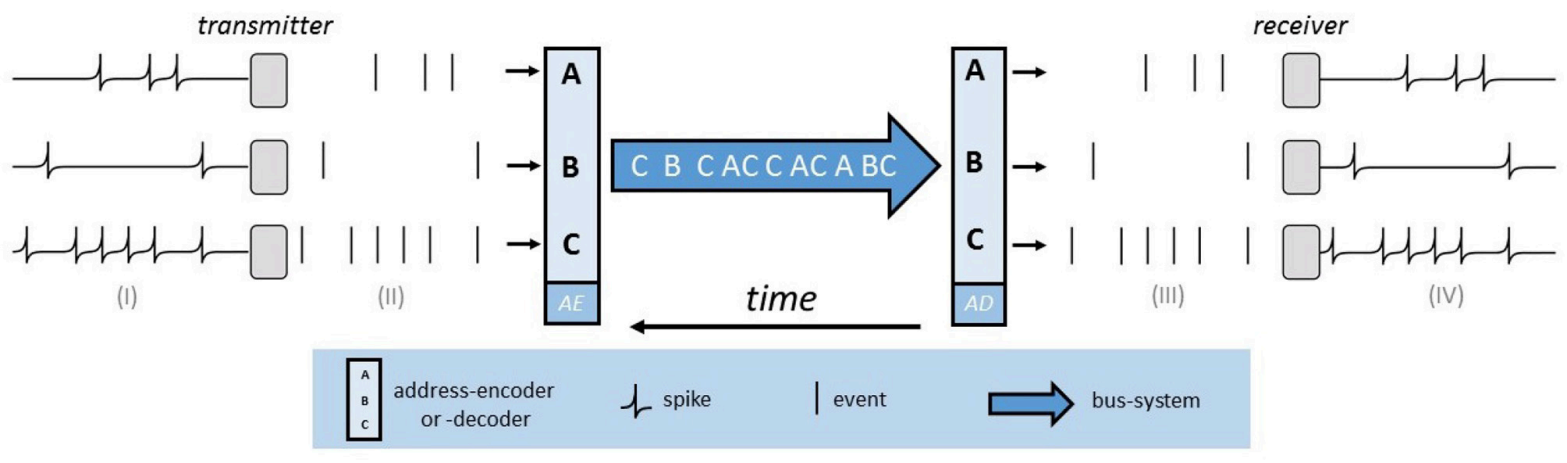
The AER-bus-system. Three neurons on the sending chip produce spikes [see (I)]. These are interpreted as binary events [see (II)] and by means of the address encoder (AE), a binary address is generated. This address is transmitted via the bus-system and the address decoder (AD) determines the correct position on the receiving chip [see (III)]. Hence a spike is emitted on the affected neuron of the receiver [see (IV)].

The basic functionality of AER is implemented, as illustrated visually in Figure 5, by two address encoder and a digital bus. The bus system implements a multiplex strategy so that all neurons and pixels transmit their information, time-coded, on the same line. The address encoder (AE) of the sending chip generates a unique binary address for each element in case of a change. This can be either a neuron that generates a spike or a pixel on which an event (exposure change) occurs. The AER bus transmits this address at high speed to the receiving chip, whose address encoder (AD) then determines the correct position and generates a spike on the respective neuron. AER uses streams of events to communicate between chips. An event is defined as a tuple *Event*(*x, y, t, p*), whereby the pixel reference of the event is given by *x* and *y*. The timestamp is given by *t* and the polarity is represented by *p*. The polarity is either positive or negative and thus indicates whether the lighting intensity has increased or decreased. The event in question will then be displayed as an ON-event, in positive, or OFF-event, in negative case.

It is easily possible to extend this technique, since on the one hand, events from different senders can be combined, and on the other hand, forwarding to multiple recipients is feasible (Lazzaro and Wawrzynek, 1995). This means that all connection types are possible; *many to one, one to many*, and *many to many*. In addition, arbitrary connections and new connections and transformations can easily be implemented with the help of this digital address system. An important advantage is that due to the asynchronous character calculations are fast and efficient.

### 3.3. Comparison of the Best-Known Exponents: DVS—DAVIS—ATIS

All modern, event-based sensors are based on the technology introduced in section 3.1. They have independent pixels that generate asynchronous events depending on exposure changes. In addition, all sensors of this type use AER (see section 3.2) for communication. The large contrast range of these cameras is based on the logarithmic compression of the photoreceptor circuits and the local, event-based quantization (Lichtsteiner et al., 2008).

**Figure 6:**
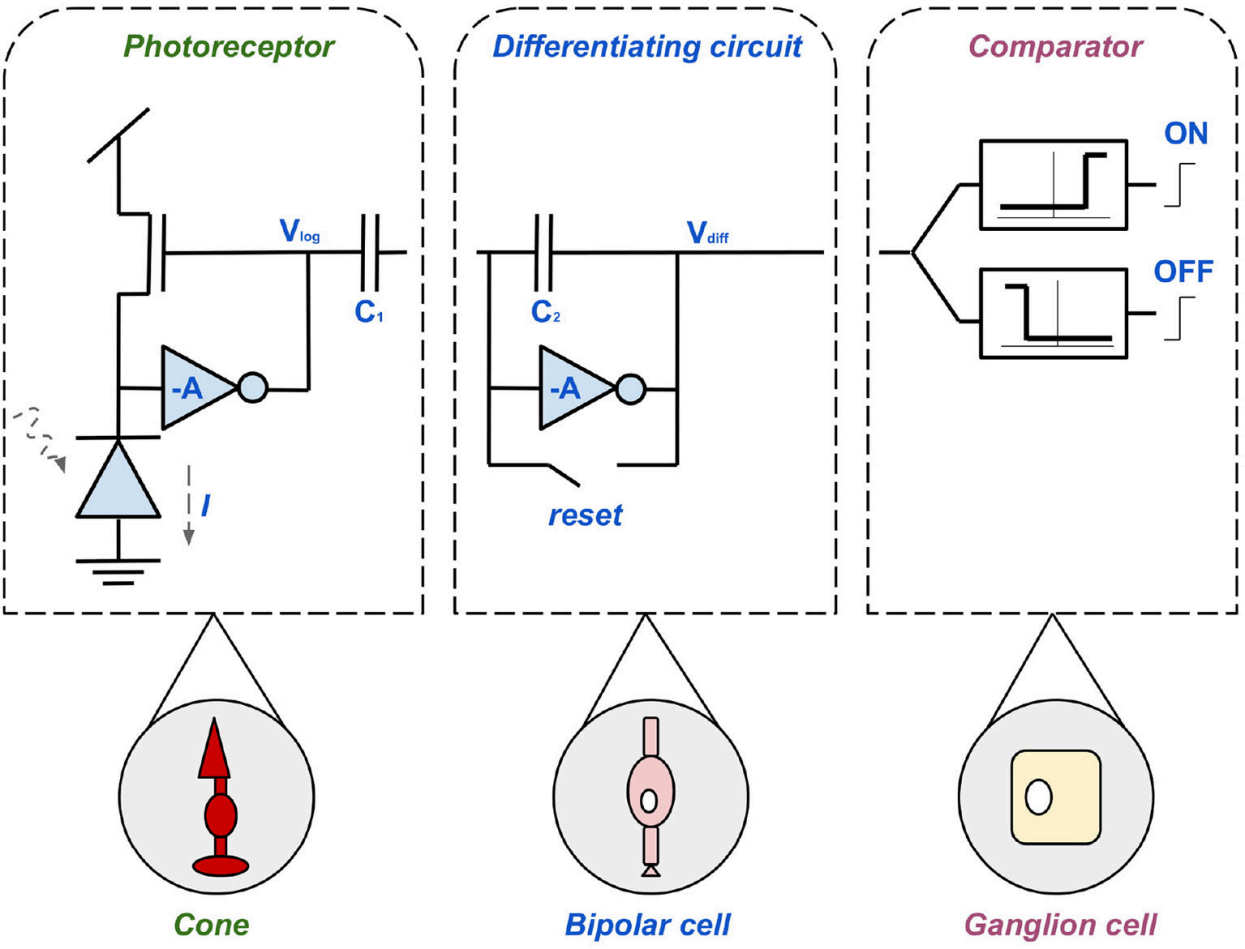
The three-layered pixel circuit of the DVS, consisting of a photoreceptor, inspired by the biological cones, a differential circuit, based on the bipolar cell and a comparator modeled after the ganglion cell.

The best known sensor of this kind, the Dynamic Vision Sensor (DVS), was developed at ETH Zurich in 2008. The circuit diagram in Figure 6 introduces the pixel design of the DVS which forms the basis of all other sensors in this section. The design decisions are based on the three main objectives; high contrast range, low error rate, and low latency (Lichtsteiner et al., 2008). To avoid unwanted oscillations there is a subdivision into sub-circuits (Brandli, 2015), as shown in Figure 6. Firstly, the left component represents the cone, a fast, logarithmic photoreceptor. Due to its logarithmic mode of operation, growth in individual pixels is effectively controlled without delaying the reaction time to exposure changes. The disadvantage of this photoreceptor set-up is that if the output is to be used without post-processing, calibration is necessary. This is due to fluctuations between the thresholds of the transistor. Secondly, the mid-component is based on the bipolar cell. Its task is to precisely amplify changes and avoid errors, generated by direct coupling (DC mismatch), through resets after each event. The third component of the DVS is the comparator consisting of two transistors. The transistors represent the ON- and OFF-ganglion cells (Lichtsteiner et al., 2008). The synergy of the components is as follows; light information is obtained by a photodiode which thus generates the current intensity *I*. Photoreceptors (cones) convert *I* into a logarithmic voltage *V*_*p*_. This voltage is inversely amplified by the factor *A* = *C*_1_/*C*_2_. Also a positive or negative event *V*_*diff*_ is generated by the differential circuit (bipolar cell), depending on the polarity of the photocurrent. Subsequently, the pulses are collected, divided into ON- and OFF-events and forwarded by the comparator (ganglion cell) (Posch et al., 2014). The logarithmic effect and the suppression of DC makes the sensor so sensitive to contrast in the time domain (Lichtsteiner et al., 2008). Hence, it takes well into account dynamic, fleeting scene information, just like biological magno-cellular structures. The functionality of P-cells, necessary for sustainable information (see chapter 2.3) is neglected (Posch et al., 2011).

**Figure 7:**
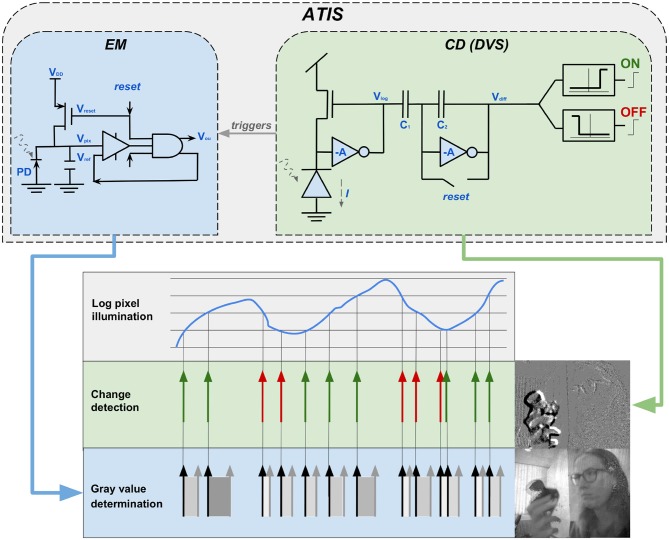
The two-section circuit constituting each ATIS pixel. The change-detector circuit (CD), which is also part of the DVS, is supplemented in the ATIS by the exposure measurement circuit (EM). In this way the camera is able to obtain, additionally to transient, also sustainable image information. Written informed consent for publication of the image was obtained from the individual in that graphic.

Posch and his team developed the Asynchronous Time-Based Image Sensor (ATIS) Posch et al. (2011). Their exponent is even closer to the biological model and also a more practically applicable sensor. The ATIS extends the basic principle of the DVS by a further photodiode to measure the time difference between two events and thus gain event-based intensity values in addition to the temporal contrast values of the event stream. As visualized in the upper part of Figure 7, the conventional change detector (CD) circuit is used to detect changes in the event stream. A circuit for exposure measurement (EM) is added. From a biological perspective, the CD-component, implemented in the DVS and the ATIS, is a magno-cellular structure. The additional EM-component embodies biological P-cells and is thus responsible to gather sustainable information. In other words, the magno-cellular CD, answers the question “*where?,”* while the parvo-cellular EM is responsible to solve “*what?*.” The application of the EM makes it possible to create gray-scale images from the events. Hereby, the intensity is given by *I* = 1/*t*, implying that the amount of the temporal difference between two events of a pixel determines its gray-level value. As visualized under *gray-value determination* in the lower part of Figure 7, a big temporal difference leads to a dark gray-value, and a small difference to a brighter one. The CD circuit triggers the activity in the EM circuit. Hence, the measurement of a new exposure time and consequently a new gray-scale value is initiated if the illumination varies (Posch et al., 2014). In Figure 7 this coherence is illustrated by the gray arrow with the label *triggers*. This process ensures that the EM circuit is also asynchronous and the corresponding gray-value is updated for each event (Posch et al., 2011, 2014; Brandli, 2015). The development of ATIS showed scientists for the first time the possibility to combine frame-based with frame-free approaches to obtain static and dynamic image information in parallel. The resulting duality also opens up a large number of new processing capabilities, since many conventional machine vision algorithms do not work with asynchronous event streams. The special design and operating principle of the ATIS also offers further advantages, some of which have direct applications; for example, video compression at sensor level can be achieved by suppressing temporal redundancies. In addition, the extremely high temporal resolution and the dynamic range of 143 dB are remarkable. ATIS owes its wide dynamic range to the fact that it encodes its intensity values time based. Conventional sensors use fixed integration times for the complete array and are thus highly dependent on light levels. Time based encoding naturally leads to separate integration times for each pixel implying the wide dynamic range and a more light independent sensor. However, this leads to uneven exposure times, which causes problems with near and slow objects (Posch et al., 2014).

**Figure 8:**
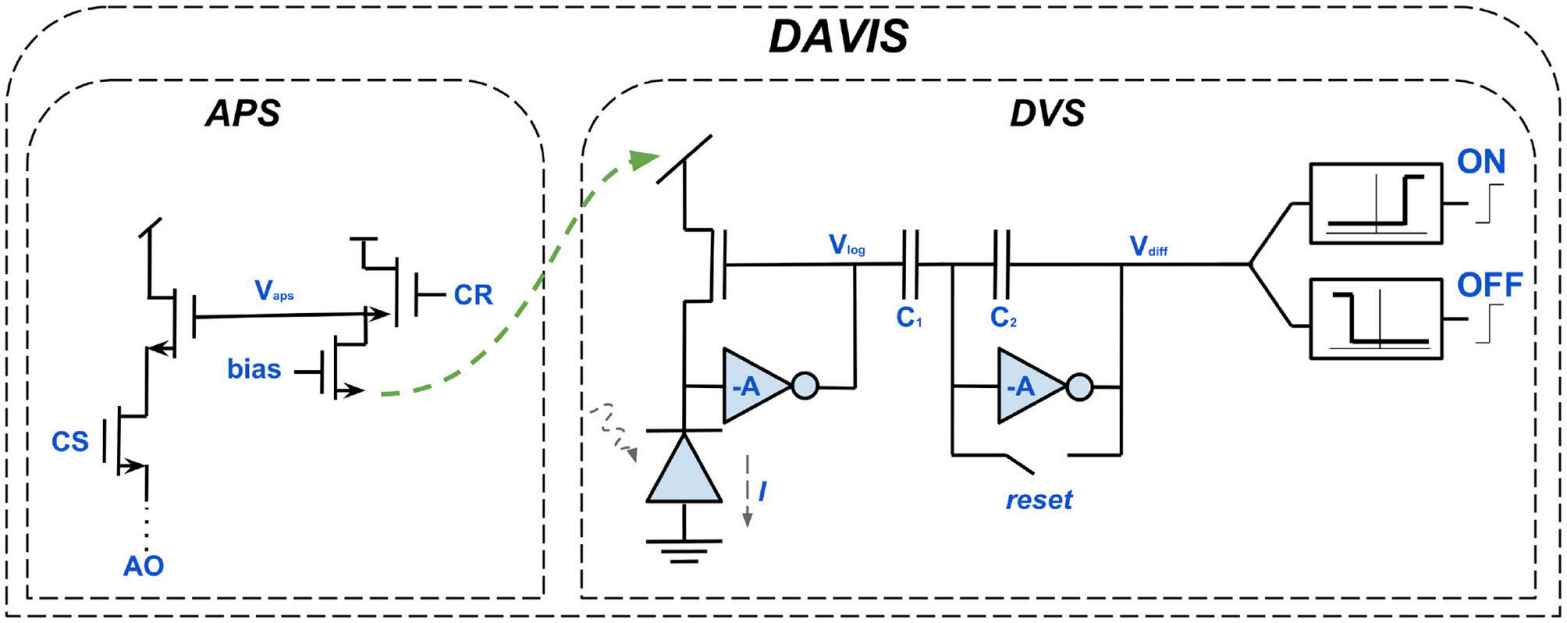
The circuits building up the DAVIS. Each of its pixels is a hybrid of the DVS-circuit and an active-pixel-sensor (APS). Like the ATIS sensor, the additional component of the DAVIS is capable of generating gray-scale images. However, the principle of operation of the APS is synchronous and thus similar to conventional vision sensors, distinguishing both sensors severely.

It was in this context that the motivation for developing the Dynamic and Active-pixel Vision Sensor (DAVIS) came about. Besides the DVS and ATIS it is the third major event-based sensor. DAVIS, introduced in Berner et al. (2013), is a hybrid of DVS and APS. As shown in Figure 8, the DVS-circuit, responsible for the asynchronous detection of logarithmic intensity changes, for generating dynamic scene information, is supplemented. Thus, all three modern event-based cameras have the same circuit as a basis. The second component of the DAVIS is an APS and, similar to the EM of ATIS, responsible for absolute exposure measurement and generating gray-scale images in addition to the event stream. In contrast to ATIS, however, the additional component of DAVIS is not asynchronous. The APS circuit receives static scene information by frame-based sampling of the intensities. This makes it very close in its operating principle to the APS component of conventional cameras. The obvious advantage of being able to use decades of research, is impaired by the existing disadvantages of frame-based cameras, such as redundancy, high latency etc. (Berner et al., 2013; Posch et al., 2014; Cohen et al., 2017).

Table 2 takes all three sensors into account. In addition to technical criteria, such as resolution, pixel size and dynamic range the costs and fields of application are also regarded. DVS is the predecessor of the other two sensors. It is the smallest and least expensive one, but has clear disadvantages in comparison. For example its low resolution of 128 × 128, its noise problems and its inability to generate intensity or gray-value information. The other two sensors each have different strengths and weaknesses. The ATIS convinces with a contrast range of 143 dB, caused by not mapping intensity values to a fixed voltage range. However, this also leads to uneven exposure times and thus to motion artifacts. The DAVIS has less problems with motion artifacts because it uses even exposure times. The synchronous mode of operation of the DAVIS, for intensity measurement and gray-value images, causes a large redundancy but also leads to independent processes which do not interfere with each other. ATIS has the highest resolution of 304 × 240, which offers considerable advantages in confusing scenes with many objects. Since the DAVIS has smaller pixels it better represents fine granular image areas with a high level of detail. As a result, ATIS is better suited for monitoring and the DAVIS for dynamic scenes with fast movements. ATIS, due to its completely asynchronous character and the fact that the theoretical basis of P-cells is taken into account, is superior in biological plausibility. DAVIS leads in practical applicability. On the one hand this is due to their small pixel size, but on the other hand also because it is better at handling darkness and near, slow objects.

### 3.4. Additional Models of Event-Based Sensors

Alongside the three best-known representatives of neuromorphic cameras, discussed in chapter 3.3, there are other models worth mentioning^1^. In 2007, long before the development of the ATIS and virtually at the same time as the DVS was created, Christoph Posch, a co-developer of DVS and ATIS, invented the Dynamic Line Sensor (DLS). This sensor was presented in Posch et al. (2007) and is quite unusual; its resolution is 2 × 256 and thus it consists of only two series of pixel. Despite this characteristic, which makes it a niche solution, the sensor has interesting properties. For example, its pixel size is with 15*μ*m smaller than that of all the sensors presented in Table 2. Additionally its high temporal resolution is noteworthy (Posch et al., 2007).

Color perception is a fundamental characteristic of biological vision. However, for long there have been no applicable event-based sensors implementing color vision (Delbrück et al., 2010; Posch et al., 2014). Experiments in this direction suffered from weak color discrimination (Berner and Delbrück, 2011; Leñero-Bardallo et al., 2014) and also had either extremely large circuits (Berner and Delbrück, 2011) or large pixels (Leñero-Bardallo et al., 2014). This was fundamentally different with the color dynamic and active-pixel vision sensor (C-DAVIS) from Li et al. (2015). It combines slightly modified pixel circuits of the DAVIS with a special type of Bayer sensor, a photosensor with color filter. The C-DAVIS generates in parallel synchronous color images and asynchronous event streams that do not receive color information. Thus, the coloring is created in the conventional part of the camera.

EBS have been continuously improved in the last years. Alongside research institutes, private companies, like Prophesee, Samsung, and HillHouse, contributed to this progress. Therefore, some of the newer models are less accessible to academia. With Samsung's DVS, a representative of neuromorphic cameras, constructed outside the academic world, was introduced for the first time in Son et al. (2017). The motivation was clearly make EBS marketable. The developers focused on reducing the pixel size to 9 *μ*m and lower the energy consumption (Yaffe et al., 2017). This VGA dynamic vision sensor embodies a digital as well as an analog implementation. The resolution was increased to 640 × 480 and AER was extended to G-AER (Group Address Event Representation) to compress data throughput. G-AER handles massive events in parallel by binding the neighboring 8 pixels into a group. This technique allows easier control of pixel biases and event thresholds (Son et al., 2017).

Another recent model is the Celex, developed at Nanyang Technological University, Singapore (NTU Singapore) (Huang et al., 2017, 2018) and distributed by the company HillHouse^2^. This sensor has a dynamic range of >120 dB and like the ATIS the Celex provides absolute brightness with every event. It is also noteworthy that the Celex IV , an event-based HD sensor is announced.

## 4. Event-Driven Stereoscopy

**Figure 9:**
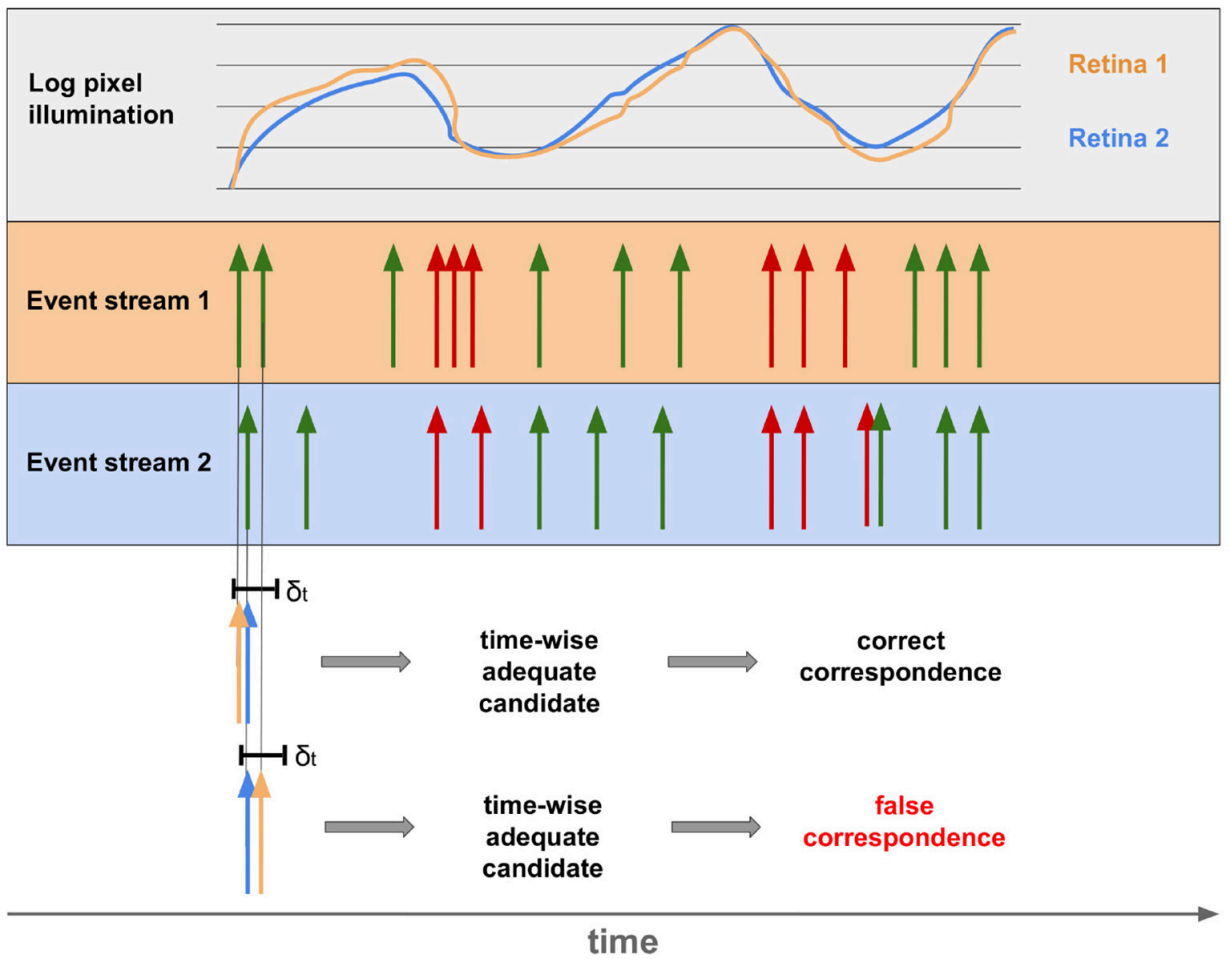
Event streams of two sensors for the same point of the real world. The top row shows the deviation of illumination between the two corresponding pixels of two retinas. The resulting streams of OFF and ON events are shown below. Below it can be seen that events of both sensors occurring within the timeframe δ_*t*_ can be both, correct and incorrect correspondences.

Although all depth stimuli depicted in section 2.4 are used in combination to enable 3D-perception in humans, binocular perception is by far the most revealing one. The other techniques sometimes provide only relative positions and are often imprecise. Binocular vision, in contrast, produces absolute values with very high accuracy (Mallot, 1998). As a result, the vast majority of event-based approaches to stereoscopy are based on binocular depth stimuli. A stereo set-up made of EBS is usually used for this purpose. To obtain disparities and thus depth information from the event streams, the individual events of both sensors must be assigned to each other. Despite the extremely high temporal resolution of EBS (Lichtsteiner et al., 2008; Posch et al., 2011), noise, faulty calibration, different contrast sensitivity of the sensors or pixels lead to deviations of several milliseconds in reality. As a result, determining matching events of both sensors with exclusively temporal aspects leads, in addition to the right ones, also to false correspondences. This is shown in Figure 9.

### 4.1. Cooperative Algorithms

To suppress false correspondences cooperative algorithms can be applied. The neurons of a SNN, that implements a cooperative algorithm, communicate according to certain rules. The research of Marr and Poggio (1976, 1977, 1979) and Marr (1982) forms the beginning of these algorithms.

To exploit the advantages of an approach based on SNN and EBS, the implementations of event-based cooperative algorithms is compatible with neuromorphic hardware. In the case of Dikov et al. (2017) and Kaiser et al. (2018) this is SpiNNaker.

#### 4.1.1. Matching Constraints

All approaches based on Marr and Poggio (1977) use two event-based sensors as input of a neural network and are therefore confronted with the correspondence problem. The naming, *cooperative algorithms*, is derived from the fact that rules are defined how the neurons of the network communicate with each other. The purpose of the communication rules is to solve the correspondence problem. Since the neurons are able to measure disparities by applying these rules, they are called disparity sensitive neurons (DSN). According to Marr and Poggio (1977), there are three steps in measuring stereo disparities: (S1) Determination of the point of interest (POI) in the first image; (S2) identification of the POI in the second image; (S3) measurement of the disparity of the two pixels. Since wrong correspondences cause problems, physical properties of solid bodies are considered in order to obtain additional constraints. These are the following two properties: (P1) is the *uniqueness* of each point in a scene at a given time. (P2) is the *continuity* of matter meaning it is continuous and divided into objects. The surfaces of objects are generally perceived as smooth (Marr, 1982). The three rules for communication between DSNs, referred to as matching constraints, are derived from P1 and P2:

- *Uniqueness Constraint (C*_1_*)*: Derived from P1, for each point of the image of the first eye/camera there is at most one corresponding hit in the image of the second eye/camera. Therefore *C*_1_ inhibits the communication between DSNs in vertical and horizontal direction (Marr and Poggio, 1979; Marr, 1982).
- *Continuity Constraint (C*_2_*)*: According to P2, physical matter is cohesive and smooth. Hence, *C*_2_ has a stimulating effect when it comes to communication in diagonal direction with the same disparity. So if a disparity of neighboring neurons is consistent, it is more likely to be correct and the corresponding signal is thus amplified (Marr and Poggio, 1979; Marr, 1982).
- *Compatibility Constraint (C*_3_*)*: Is derived from the thesis of Marr and Poggio (1977), that *black dots can only match black dots*. It states that similar characteristics in the same region are more likely than completely different ones. In practice, it causes ON-events and OFF-events occurring temporally and spatially dense to inhibit each other. There is a greater probability of incorrect correspondences since contrasting changes in lighting are less common for neighboring pixels (Marr and Poggio, 1977; Marr, 1982).

#### 4.1.2. Extension to Pulse-Coupled, Event-Based Technologies

The principles presented in chapter 4.1.1 are transferred into an event-based implementation in Firouzi and Conradt (2016). However, it does not use spiking neurons and is thus not exploiting the event-based data ideally. In Dikov et al. (2017) and Osswald et al. (2017), SNNs and the corresponding neuromorphic hardware are combined with this approach. Compared to conventional synchronous methods, these models use a further constraint to suppress false correspondences, *time* (Dikov et al., 2017). This brings a great novelty to the old approach; the network input is not composed of static images but instead spike-trains containing spatio-temporal information. The network implemented in Osswald et al. (2017) consists of three essential parts; retina coordinates, represented by OFF- and ON-neurons, coincidence detectors and disparity detectors. The aim is to amplify correct correspondences and to suppress wrong ones in order to generate a correct disparity measurement. The retina coordinates generate a spike for each change in illumination at a specific point in space. The random detectors signal simultaneous spikes for possible layers. The cells are arranged so that each spike represents the position of a possible disparity. Since coincidence detectors are sensitive for right but also for wrong correspondences, all possible hits are gathered here. Within the connections of the neurons of coincidence detectors and disparity detectors rules of binocular vision are implemented. In greater detail, C2 and C3 are realized by stimulating and inhibiting compounds. The uniqueness rule C1, is implemented subsequently through recurrent, inhibitory connections of the disparity detectors.

In Osswald et al. (2017), the effects of this approach are analyzed. The authors compare the spike rates of the random detectors with those of the disparity detectors. The conclusion is that wrong correspondences are detected significantly more often without matching constraints.

**Figure 10:**
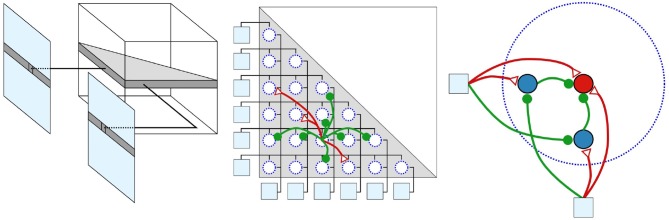
Structure of the network inspired by Dikov et al. (2017) with increasing degree of detail. The network's 3D-structure is represented in the left part; An entire row of pixels is mapped from both EBS to one plane of the SNN. This 2D-layer is where the disparities of the affected pixels are calculated. The center shows the neurons of a 2D-layer, connected according to the constraints of cooperative algorithms, outlines in chapter 4.1.1. Green represents inhibiting and blue exciting synapses. In the right part the outline of micro ensembles are visualized. The cooperative manner of this network relies on micro-ensembles. The retina coordinates are visualized as light blue squares, the blocking neurons as blue circles and the collecting neuron as a red circle. Reprinted by permission from Springer Artificial Neural Networks and Machine Learning (Kaiser et al., 2018).

In case, one of the sensors of the stereo set-up is exposed to high-frequency stimuli, false correspondences can arise. This is because the DSN, which collects the signals of both retina coordinates, exceeds its threshold, although only one of the sensors sends a pulse and the other does not. To overcome this issue, the basic technique was extended in Dikov et al. (2017) by micro-ensembles. Hereby, neuronal micro-ensembles are used that implement the behavior of a logical &. For this purpose, as shown in Figure 10, two blocking neurons are connected between the retinal coordinates and the integrating DSN. In case the left retina neuron receives a spike, it excites both, the integrating and the left blocker neuron. At the same time it inhibits the right blocker neuron. If now the right retina neuron does not receive a spike and therefore does not inhibit the left blocker neuron, this prevents the integrating neuron from generating a spike. This mechanism ensures that the integrating neuron is only capable of spiking if both blocker neurons of the ensemble are inhibited. Hence the integrating neuron only emits a pulse if both retina neurons are spiking (Dikov et al., 2017; Kaiser et al., 2018). In Dikov et al. (2017) and Osswald et al. (2017) disparities are calculated merely from dynamic scenes. This is simply to the fundamental technology of event-based cameras that perceive only changes in lighting and are thus not perceiving static scenes continuously. In Kaiser et al. (2018), this approach, including EBS, is extended to static scenes by applying synchronous microsaccades. In biology, microsaccades are extremely fast and very small eye movements with low amplitude. This artificial dynamic allows the network to extract disparities from static scenes. For the practical implementation, a robot head has been constructed which is capable of carrying out vertical and horizontal tilt movements independently and simultaneously.

#### 4.1.3. Network Architecture

On an abstract level, the network receives signals from two EBSs processing the data and extracting the disparities. The simplified structure of the SNN is as follows; events of two EBS that are on the same epipolar plane of the real world are input of the same 2D-plane of the network, as shown in Figure 10. These 2D-layers are stacked to form the 3D-SNN. Each 2D-layer calculates the disparities for a pixel row of both DVS. The neurons of the output layer generate a pulse when the corresponding point of the real world changes from occupied to unoccupied or vice versa (Dikov et al., 2017; Osswald et al., 2017; Kaiser et al., 2018).

However, the special structure of the network and its internal connections are essential for solving the correspondence problem. Therefore, the structure of the network is discussed in more detail; a disparity is indicated by a DSN that exceeds its threshold. Each DSN describes, by its x- and y-position in combination with the disparity for which it is sensitive, a specific point in space. There are two ways to arrange these neurons so that they represent the replicated scene of the real world. The naive way is that each DSN represents a point of the real world and all neurons are equally distributed, as are their real counterparts. We call this the dynamic path because the DSNs are not assigned fixed retina coordinates. For this approach, the cameras must be calibrated very accurately, their exact position and orientation must be known, and their focus line must be taken into account throughout. These conditions are difficult to implement in practice. Alternatively, each DSN represents a fixed neuron from both populations of retina coordinates. Thus same two pixels of the respective sensor are always connected with each other. This is the static method which is much less error-prone.

### 4.2. Extensions of Cooperative Algorithms

The formalisms of cooperative algorithms already eliminate the majority of false correspondences. However, some scientists found ways to combine this basic approach with an entirely different approach in order to obtain even more accurate results. In the following methods are shown that complement cooperative algorithms.

#### 4.2.1. Gaborfilters

In Camuñas-Mesa et al. (2014a,b) and Reverter Valeiras et al. (2016), the authors show how to extend cooperative algorithms extended by a new component, a supplementary constraint. On top of the known matching constraints such as time and polarity, the authors use Gabor filters to extract information about the object edges that generate the events. This is working well because EBS create events when objects, and thus their edges, move. Events that belong together refer to the same edge and should therefore have the same orientation.

The authors of Camuñas-Mesa et al. (2014a) use Gabor filters, with different angles and scales, on the original event streams of both cameras. The results are used as input for a cooperative algorithm.

The work of Reverter Valeiras et al. (2016) is based on the *HFirst-approach* of Orchard (Orchard et al., 2015), which uses a hierarchical, h-max-inspired SNN-architecture and a neuromorphic camera for object recognition. In Reverter Valeiras et al. (2016), an ATIS (see chapter 3.3) is applied. The approach describes itself as actually event driven, as each new event renews the 3D-pose estimation.

#### 4.2.2. Belief Propagation

A new completely event-based approach to depth perception is presented in Xie et al. (2017). It is based on *Belief Propagation* (BP), a subset of the *message-passing* algorithms. These algorithms are used to solve derivation, optimization and condition fulfillment problems. Bayesian networks or Markov Random Fields are often applied for preprocessing in this context. BP then calculates the marginal distribution of each unobserved node. Hence, the correspondence problem is seen as a labeling problem. The labels refer to the disparity and their quality is measured as a cost function. By use of maximum a posteriori probability (MAP), labels are determined that minimize the cost function. The method consists of four steps; preprocessing, adjustment, Belief Propagation, and output of disparities. The pre-processing consists of noise filtering and correction of the input images. For correction it is transformed so that each pixel row of the two images refers to the same points. The matching determines whether two events are potential partners. Correct matches are from different sensors, occur within a time window, have the same polarity and occur in the same or adjacent rows. This implementation of Belief Propagation is based on Felzenszwalb and Huttenlocher (2004). The algorithm does not synchronously renew all disparity estimates, but always the neighborhoods of new events. The output of the algorithm is a *belief vector* for each node. The label, thus the disparity, is then chosen so that it minimizes the cost function.

Kogler, who tried for a long time to apply classical algorithms to event-based data, offers in Kogler et al. (2014) an alternative realization of event-based stereo vision with Belief Propagation. He complements this with a subsequent filtering in two phases.

#### 4.2.3. Combining Spatial and Temporal Aspects With Luminance and Motion

In the discussed approaches of this chapter, as well as in chapter 4.1, the correspondence problem is commonly solved by spatial constraints and luminance. According to the authors of Ieng et al. (2018), disparities are thus not reliably detected in uncontrolled lighting conditions and unstructured scenes. Therefore, Ieng et al. (2018) presents an entirely time-based method that exploits the unique characteristics of neuromorphic sensors, such as high temporal resolution, even more. For this purpose, the ATIS presented in chapter 3.3 is applied, which in addition to change events also uses the luminance encoded in the form of temporal differences. This approach does not represent a completely new method, but rather an extension of known approaches. Hereby, the precise timing of Kogler et al. (2011a) is combined with the local motion consistency of Benosman et al. (2012) and the temporal consistency of the luminance from Posch et al. (2011). By additional luminance information wrong correspondences are reduced. This means that the unique principle of operation of the ATIS, which in contrast to DAVIS works completely asynchronously, leads to new results. Due to the consideration of many different approaches and theoretical considerations, the algorithm is extremely complex. Spatial, temporal, generalized temporal, and motion criteria are combined.

### 4.3. Alternatives to Cooperative Algorithms

The approaches presented so far (see chapter 4.1 and 4.2) are all based on the biological theories of binocular vision investigated by Marr and Poggio (1979). An alternative implementation of a cooperative network compatible with their research is given in Piatkowska et al. (2013). Piatkowska developed an adaptive, cooperative algorithm adjusting the disparity estimation with each new event. In Piatkowska et al. (2017), the approach is enhanced and the error rate, determined by MME, can be reduced by 50%. For this purpose the normalization is altered and a noise filter is used. The authors also surrogate the applied DVS through ATIS.

This chapter introduces other methods for stereo viewing with event-based cameras, beside cooperative algorithms.

#### 4.3.1. Conventional, Perspective-Based, and Active Techniques

In Schraml et al. (2007), a conventional, area-based approach to solving the correspondence problem is transferred to event-based cameras. Area-based approaches use the neighboring pixels to find correspondences between the two images for groups of pixels. The authors tested classical cost functions such as Normalized Cross-Correlation (NCC), Normalized Sum of Absolute Differences (NSAD), and Sum of Squared Differences (SSD). It is questionable whether it makes sense to implement such an algorithm with EBS because the pre-processing consists of reconstructing gray-value images from the events. In addition, such a classical algorithm was compared by Kogler in Kogler et al. (2011a) to a time-based approach and did much worse, especially because of its error rate of 4.91%. In Kogler et al. (2009), the area-based approach is combined with a feature-based approach for EBS. This work, combining classical algorithms with event-based technology, is also pursued in Dominguez-Morales et al. (2011) and Belbachir et al. (2012). The researchers around around Kogler, however, state in Kogler et al. (2011b) that classical approaches to stereoscopic vision do not take account the advantages of silicon retinas and that the reconstruction of images leads to a loss of temporal accuracy. Based on this consideration, an algorithm is developed in Kogler et al. (2011b) focusing on the temporal correlation of events. This approach is considered by the developers themselves to be far superior to their previous experiments.

A separate class of algorithms for event-based stereoscopy are the perspective approaches (Benosman et al., 2011; Rogister et al., 2012), which are to be clearly separated from the classical methods and often serve as a basis for advanced algorithms. Epipolar geometry is used as a constraint in order to allow to reconstruct 3D-structures. Events are reconstructed, within a time window, and based on their distance to the epipolar line. Wrong correspondences are additionally eliminated by considering polarity, orientation and order. In Carneiro et al. (2013), this is enhanced by applying a Bayesian filter.

Quite different solutions to recover depth from event-based data are shown in Martel et al. (2018) and Haessig et al. (2019). These are active techniques and require additional hardware, setting them apart from most investigated methods. In Haessig et al. (2019), the known method to estimate depth from the amount of defocus at the focal plane is transferred to neuromorphic sensors and hardware. This is a simple yet elegant solution, whereby the camera alters its focal distance in a steady manner and a SNN computes the time of the best focus for each pixel, creating a depth map. This approach requires a specific liquid lenses, as an adjustable focal distance is necessary to allow a variable focus. According to the authors the low power consumption and computation times guarantee a real-time application. Complementing the event-based stereo setup, two mirrors and a mirror-galvanometer driven laser are used in Martel et al. (2018). This equipment allows the creation of *light spots* in the scene, where contrast varies a lot. Two DAVIS capture these contrast changes, detecting the laser-induced events enabling a resource-efficient matching. Events are clustered by space-density, using a simple mean-shift algorithm, high-density filter and triangulation using a direct linear transform in the overlap field of both sensors. A rare feature of this method is that sensor synchronization is not required.

#### 4.3.2. Event-Based 3D-Perception Through Monocular Cues

In Rebecq et al. (2017), Rebecq presents a method for Event-based Multi-View Stereo (EMVS). The approach is based on *dense* methods (see chapter 2.2) for conventional cameras. These approaches, determine dense 3D-structures from known angles. EMVS, which is based on the work of Space-Sweep Approach (Collins, 1996), estimates semi-dense 3D-structures with only one event-based camera. The camera is thereby moved on a known trajectory. The moving sensor obtains edge detection and continuous measurement data. The algorithm comprises three sub-steps; (1) events are projected back in the form of beams. (2) These beams are counted in a voxel grid to measure the spatial density of the beams. (3) A semi dense reconstruction of the edges is possible due to local maxima. A unique characteristic of this approach is that only one sensor is used for depth perception and no additional aids are applied. Also, the camera is not fixed but moves on a given trajectory. The authors report that their method handles noise, fast movements and poor lighting well.

Further approaches to monocular depth perception are presented in Brandli et al. (2014) and Matsuda et al. (2015). These methods distinguish themselves from EMVS by using complementary hardware and not relying merely on the data of one camera. In Brandli et al. (2014), a pulsed line laser is used in railing reconstruction whose asynchronous operating principle can be easily combined with an event-based algorithm. In Matsuda et al. (2015), the EBS is supplemented by Structured Light.

## 5. Conclusion

Neuromorphic systems have enormous potential, yet they are rarely used in a non-academic context. Particularly, there are no industrial employments of these bio-inspired technologies. Nevertheless, event-based solutions are already far superior to conventional algorithms in terms of latency and energy efficiency. Potential consequences and the future of such technologies and processes are discussed in this chapter.

### 5.1. Remaining Issues of Silicon Retinas

Although much research with biologically inspired sensors has taken place in recent decades, there are still plenty of unresolved issues and open questions in the field. Techniques based on Mahowald's VLSI-retina are in some respects quite similar to the structures of the human brain and eyes, which they are imitating. At the same time, however, there are many biological mechanisms and structures that are not, or only partly, implemented artificially. A popular example of this is the *wiring problem* in biological neural 3D-structures (Posch et al., 2014). Although 3D-wiring has been regarded as the more efficient technology for more than 20 years (Milenkovic and Milutinovic, 1998), there are still only a few immature approaches (Kurino et al., 2000; Culurciello and Andreou, 2005).

Another fundamental feature of biological vision is color perception. C-DAVIS, a neuromorphic sensor capable of color recognition, has been available since 2015, but color perception is only implemented in the synchronous and not in the event-based part of the camera (Li et al., 2015). Sensors that encode color information in the events do not yet exist. However, it can be argued that color and motion perception is also processed separately in biological vision, by means of cones and rods, and therefore a division of the mechanisms is justified.

Furthermore, the problem of spatial redundancies and how they can be effectively reduced remains unsolved. Moreover, even the relatively high resolution of DAVIS and ATIS is much too small for industrial purposes and their already strongly reduced pixel size are still too large (Posch et al., 2014).

### 5.2. Artificial Stereoscopy—A Comparison

The comparatively low dynamic range and the limited frequency of conventional camera systems form a bottleneck for classical approaches to stereoscopy. In addition, these methods are very unreliable under uncontrolled lighting conditions. In Akolkar et al. (2015), the advantages of event-based sensors for pattern recognition are discussed in detail. This can essentially be transferred to stereoscopy. Motion artifacts and object displacement of synchronous image acquisition are the reason why asynchronous imaging is sophisticated in stereo vision.

However, the use of EBS is not sufficient, which is indicated by the fact that the algorithms have very different results. For example, approaches based on classical methods for stereo vision cannot compete with cooperative algorithms. The authors, of the methods introduced in section 4.1, state that range-based and feature-based approaches have significantly worse results than simple algorithms using temporal correlation. This is especially interesting since temporal correlation is only the most basic criterion of the cooperative algorithms in chapter 4.1. Cooperative algorithms are the gold standard, which can also be seen by the fact that they have been used successfully by several independent research groups (Dikov et al., 2017; Osswald et al., 2017; Piatkowska et al., 2017; Kaiser et al., 2018). The approach of section 4.2.3 introduced in Ieng et al. (2018) was published in summer 2018 and is therefore quite new. In addition, it builds on many previous works. As a result, it is very progressive and combines many benefits of the research it is based on. Also noteworthy are the results of Martel et al. (2018) and Haessig et al. (2019). Hereby, active approaches requiring additional hardware are introduced. However, these techniques are resource-efficient allowing a real-time application.

### 5.3. Outlook

Algorithms are based on SNNs and EBS only develop their potential when they are applied on neuromorphic hardware (Khan et al., 2008). Although there are already some implementations of networks on neuromorphic hardware (Dikov et al., 2017; Andreopoulos et al., 2018; Kaiser et al., 2018), research in this area is not that far yet. However, this will probably change rapidly in the next few years which will make the existing approaches much more powerful.

The application of DAVIS or ATIS in contrast to DVS has already significantly improved the outcome of several approaches like (Reverter Valeiras et al., 2016; Piatkowska et al., 2017; Andreopoulos et al., 2018; Ieng et al., 2018). In particular responsible for this progress is the higher resolution of these sensors. Even though solutions from industry, such as Samsung's DVS (Son et al., 2017), are not yet mature, this could alter drastically within the next few years. A likely consequence is that the costs of these cameras will decrease. This development is further strengthened by the fact that several scientists, which had an important part in the development of DVS, DAVIS and ATIS, are transferring their expertise to the industry by founding companies. Examples for this are Insightness^3^, Prophesee^4^, and iniVation^5^. This trend suggests that many problems of current algorithms can be solved by better sensors and respective technologies.

## Author Contributions

LS, DR, AR, and RD are responsible for the idea, the core concept, and the architecture of this paper. LS, JW, DR, and JK did the research and wrote the paper.

### Conflict of Interest Statement

The authors declare that the research was conducted in the absence of any commercial or financial relationships that could be construed as a potential conflict of interest.

## Footnotes

^1^An overview of neuromorphic sensors and their applications is given here: https://github.com/biphasic/event-based_vision_resources

^2^More information about HillHouse: http://www.hillhouse-tech.com

^3^More information about Insightness: http://www.insightness.com

^4^More information about Prophesee: https://www.prophesee.ai/

^5^More information about iniVation: https://inivation.com
